# Primary health care: realizing the vision

**DOI:** 10.2471/BLT.20.279943

**Published:** 2020-11-01

**Authors:** Shannon Barkley, Robert Marten, Teri Reynolds, Edward Kelley, Suraya Dalil, Soumya Swaminathan, Abdul Ghaffar

**Affiliations:** a Integrated Health Services, Universal Health Coverage and Life Course, World Health Organization, Avenue Appia 20, 1211 Geneva 27, Switzerland.; b Alliance for Health Policy and Systems Research, Science Division, World Health Organization, Geneva, Switzerland.; c Primary Health Care Programme, World Health Organization, Geneva, Switzerland.; d Science Division, World Health Organization, Geneva, Switzerland.

Primary health care is the cornerstone of a strong health system and accelerates progress towards universal health coverage (UHC) and the sustainable development goals (SDGs). A primary health-care approach includes primary care and essential public health functions at the core of integrated health services, empowered people and communities, and multisectoral policy and action. The global political commitment to this approach was codified in the 2018 Declaration of Astana and reiterated in the 2019 Political Declaration of the High-Level Meeting on Universal Health Coverage. In 2020, the coronavirus disease 2019 (COVID-19) pandemic demonstrates that gaps in primary health-care implementation have weakened countries’ abilities to detect and respond to the outbreak, and to keep essential health services functioning. Primary health care must feature in health system efforts to build back better.

Evidence demonstrates that a primary health-care approach is the most equitable, effective and efficient way to improve health. Primary health-care implementation has evolved over time to address demographic and economic challenges as well as shifting disease burdens. Research has informed this evolution and continues to highlight what works to strengthen health systems aligned to primary health care. Yet in many settings, persistent gaps in contextualized knowledge, capacity for research and locally acceptable solutions exist – and are exacerbated by a lack of political will, insufficient leadership and inadequate funding.

This theme issue helps fill these gaps. The articles present evidence, strategies and practical implementation considerations for policy reforms and health system transformations needed to attain UHC through primary health care. Articles consider various aspects of the science and practice of primary health care: an examination of the primary health-care approach after the Declaration of Astana and in the context of COVID-19,^1^ the potential and limitations of primary health care to contribute to the SDGs,^2^ the role of communities in primary health care and UHC,^3^ the role of primary health care in addressing the climate crisis,^4^ a call for global health donors to reorient their financial support towards primary health care^5^ and the testing of a monitoring framework for primary health care.^6^ The issue also highlights issues of integrated health service delivery, focusing on primary care: utilization patterns for health services that can be delivered in primary care,^7^ the role of pharmacists in primary care delivery,^8^ the delivery of essential surgical services by family doctors,^9^ important linkages between primary care and emergency, critical and operative care,^10^ the role of primary care in addressing the social determinants of health^11^ and strategies to retain rural health workforce.^12^ The issue further includes description of country progress towards primary health care, including Ethiopia’s implementation of primary health care towards UHC, and a synthesis of twenty country case studies.^13^

The call for papers for this theme issue closed just 15 days after the recognition of COVID-19 as a Public Health Emergency of International Concern. The current COVID-19 pandemic, where poor, vulnerable, older people and those with chronic disease are at the highest risk of illness and death, continues to underscore the need to strengthen the three components of primary health care. Primary health care emphasizes the role of public health functions (including health promotion, health protection, disease prevention and surveillance and early warning mechanisms) and primary care. Robust primary care enables services to be delivered as close to people as possible and ensures appropriate connections between service delivery platforms, thus promoting early identification, safe referral and timely care for people affected by COVID-19 as well as enabling continuity of essential health services for acute and chronic conditions. Doing so reduces both direct morbidity and mortality from COVID-19 and indirect morbidity and mortality. A primary health-care approach emphasizes the complementary and interdependent roles of public health officials, health workers and communities in emergency preparedness and response. The emphasis of primary health care on multisectoral action for health promotes a multisectoral response in an emergency context.

Primary health-care implementation through multisectoral and health sector policies, strategies and operational plans and health service delivery should be informed by evidence of what works and how. Health systems and implementation research on interventions that support all three components of primary health care is key to providing this information, and this requires adequate and sustainable allocation of funds and human resources for primary health-care oriented research. This theme issue, other similar dedicated journals and issues on primary health care, as well as emerging dedicated research consortia and efforts are helping fill evidence gaps. Much more context-specific information, complemented by the analytic and implementation capacity to apply these learnings, is needed to drive evidence-informed health sector and health service transformation towards health and well-being for all.
